# Tuberculosis: A Cunning Disease Presenting with Endopericarditis- Associated Bilateral Uveitis

**DOI:** 10.4274/tjo.galenos.2019.55889

**Published:** 2019-12-31

**Authors:** Gholam Hossein Yaghoubi, Farshid Abedi, Masoud Ziaee, Amir Norouzpour

**Affiliations:** 1 Birjand University of Medical Sciences, Department of Ophthalmology, Birjand, Iran; 2 Birjand University of Medical Sciences, Department of Infectious Diseases, Birjand, Iran; 3 Shiraz University of Medical Sciences, Poostchi Ophthalmology Research Center, Shiraz, Iran

**Keywords:** Tuberculosis, endophthalmitis, endocarditis, pericarditis, polymerase chain reaction (PCR)

## Abstract

*Mycobacterium tuberculosis* can spread through the entire body but rarely involves the eye. We report a patient with endophthalmitis in one eye and simultaneous retinal vasculitis in the fellow eye. Systemic work-up suggested infective endopericarditis. Polymerase chain reaction analyses of the vitreous and pericardial fluid were positive for *M. tuberculosis*. We initiated a four-drug antituberculous treatment regimen (isoniazid, ethambutol, pyrazinamide, and rifampin). After two weeks, we discontinued all the medications due to drug-induced hepatitis. We restarted isoniazid and rifampin, but hepatitis recurred. Finally, we chose isoniazid/ethambutol combination for 18 months, and also administered short-term systemic corticosteroid. His vision improved considerably with no recurrence of hepatitis or tuberculosis for 3 years after completion of treatment. Ocular tuberculosis can masquerade as other causes of intraocular inflammation, and a medical team consisting of an ophthalmologist and an infectious disease specialist might be needed for the diagnosis and management.

## Introduction

Tuberculosis (TB) is a noticeable public health problem with an increasing incidence in recent years. TB can either be restricted to one organ, most commonly the lung, or spread throughout the body, involving multiple organs.^1^ Ocular TB is a rare presentation of extrapulmonary TB which accounts for 0.2-18% of TB cases, depending on the geographic area.^2^ Almost all parts of the eye can be involved, but the most common manifestations are chronic uveitis, choroiditis, and keratitis.^3^ It occasionally mimics intraocular malignancies or other causes of ocular inflammation.^4^ It needs a high clinical suspicion to be diagnosed as early as possible before any permanent visual loss occurs. In this case report, we present a patient with endogenous endophthalmitis in one eye and retinal vasculitis in the fellow eye, most probably resulting from *Mycobacterium tuberculosis-*induced infective endopericarditis.

## Case Report

**Figure 1:**
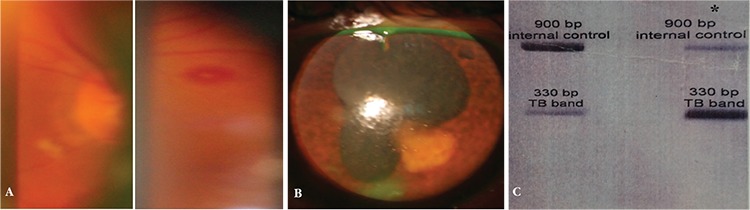
Clinical examination findings and polymerase chain reaction (PCR) results of the patient. A) Fundus examination of the right eye shows retinal vasculitis and a Roth spot which are distributed over the entire retina. B) Slit-lamp biomicroscopy of the left eye shows corneal stromal edema, intense anterior chamber reaction, posterior synechiae, and an iris granuloma close to the posterior synechia. C) PCR result from vitreous tap was positive for M. tuberculosis. The positive control is in the right column, shown with an asterisk sign (*)

A 45-year-old man presented to our hospital with subacute low-grade fever, malaise, and myalgia. A few days after admission for sepsis work-up, the vision in both his eyes gradually blurred within a few days of each other. His medical and ocular history was unremarkable. Visual acuity was 20/400 in his right eye and 20/630 in the left eye. The right eye had moderate nuclear sclerosis, retinal vasculitis, and Roth spots (Figure 1a). The left eye had ciliary injection of the conjunctiva, corneal stromal edema, dense cells and flare in the anterior chamber, and posterior synechiae (Figure 1b). The vitreous was hazy, and a blurred view of the fundus without retinal detachment was obtained. Endogenous endophthalmitis in the left eye was suspected; therefore, vitreous aspiration as well as intravitreal vancomycin (1 mg/0.1 mL) and amikacin (0.4 mg/0.1 mL) injection were performed. Vitreous, blood, and urine cultures were all negative, but vitreous polymerase chain reaction (PCR) analysis was positive for *M. tuberculosis* (Figure 1c). The results from a complete systemic work-up were positive for an elevated erythrocyte sedimentation rate and C-reactive protein, but negative for HIV infection. A chest roentgenogram showed no significant pathologic changes at initial presentation. Whole-body bone scintigraphy was noncontributory other than mild right sternoclavicular arthritis. Echocardiography showed a large (15x15 mm) mobile mass in the left atrial side of the mitral valve with severe mitral regurgitation. Infective endocarditis was suspected, and mitral valve replacement surgery was performed. Pericardial fluid had high levels of lactate dehydrogenase and adenosine deaminase with normal levels of protein and glucose. Pericardial fluid PCR was positive for *M. tuberculosis*, but culture was negative. Pericardial biopsy showed chronic fibrohistiocytic reaction with no dysplastic changes. A four-drug antituberculous treatment regimen (isoniazid, ethambutol, pyrazinamide, and rifampin) was started. After two weeks, the patient exhibited clinical signs of hepatitis, and serum aspartate aminotransferase and alanine aminotransferase levels were elevated up to four times higher than normal. We discontinued all the anti-TB medications, after which the hepatitis resolved. We restarted only isoniazid and rifampin, but the hepatitis recurred. We discontinued the medications again until hepatic enzyme levels returned to within normal range. Serology for viral hepatitis was negative. We then chose the combined isoniazid/ethambutol regimen. There was no recurrence of hepatitis this time, and liver enzyme levels remained within normal range. We administered short-term systemic corticosteroid to reduce the risk of mortality and prevent progression to constrictive pericarditis. His vision returned to 20/25 in the right eye and 20/32 in the left eye two months after the treatment was initiated. The two-drug regimen was continued for 18 months. No systemic relapse has occurred during the intervening 3-year follow-up period, and his vision has remained unchanged.

## Discussion

*M. tuberculosis* can involve any organ, but most commonly affects the lung. It can also involve any part of the eye. TB can invade the eye either as a primary or secondary infection. Primary infection is usually limited to the conjunctiva and cornea, but secondary infection is more widespread, resulting from either contiguous spread from an adjacent tissue or hematogenous spread. The most common ocular manifestations in secondary infections are chronic uveitis, choroiditis, and keratitis.^3^ Panophthalmitis, endophthalmitis, and vitritis have also been reported.^5^ Here, we presented a case with endophthalmitis in one eye and simultaneous retinal vasculitis in the fellow eye.

Diagnosis of ocular TB is a challenging issue for ophthalmologists.^2^ A negative smear for acid-fast bacilli, failure to culture the bacilli, and lack of necrotizing granulomas on histopathology specimen do not, however, exclude the diagnosis of TB. The tuberculin skin test, interferon gamma release assays, and chest roentgenograms might not be helpful for diagnosis of ocular TB.^6^ PCR has been a robust diagnostic technique particularly for ocular TB since it requires only a small sample with no need for the cells to be viable.^7^ Although vitreous culture was negative in our case, vitreous PCR was positive for *M. tuberculosis. *

TB endophthalmitis is a rare intraocular inflammation which usually results from hematologic spread of a lung or central nervous system infection.^5^ However, the pulmonary foci might not be clear clinically or radiographically. It has been reported that even up to 60% of extrapulmonary TB might not have pulmonary disease.^8^ In our case, we did not find a primary source for TB infection other than the heart. Serial chest roentgenograms did not show any remarkable changes typical of TB infection other than mild pulmonary edema with pleural effusion. Pleural as well as cerebrospinal fluid PCR analyses were negative for *M. tuberculosis*. On the other hand, TB has been reported to affect the pericardium,^9^ myocardium,^10^ endocardium, and valvular structures.^11^ In our case, the ocular manifestations were most probably secondary to hematologic spread of infective endopericarditis. Pericardial fluid PCR was positive for *M. tuberculosis*, supporting our hypothesis.

The main therapeutic challenges in TB infections are patient compliance and the increasing incidence of drug resistance. Because TB is endemic in Iran, the prevalence of drug resistance might be high. A bacteriologic sensitivity test could help us to find the most appropriate therapeutic regimen for our case; however, we empirically chose the four-drug regimen (isoniazid, ethambutol, pyrazinamide, and rifampin) for the initial phase. This antibiotic combination has been effective in most TB cases in our general hospital. Systemic corticosteroid administration to patients with presumed ocular TB or tuberculous pericardial effusion remains controversial.^9^ It may reduce mortality in HIV-negative patients, but it might lead to recurrence of ocular inflammation,^12^ and the effects on preventing progression to constrictive pericarditis remain obscure.^13^ We administered short-term systemic corticosteroid to our patient and obtained a favorable clinical response with no recurrence for 3 years after completion of treatment.

Response to treatment as well as drug toxicity are monitored clinically and sometimes using laboratory techniques. Our patient showed clinical improvement in the first 2 weeks after the initial regimen was started, but the occurrence of hepatitis did not allow us to continue the quadruple regimen. Hepatotoxicity is the most common adverse effect of anti-TB medications. Serum liver enzyme levels and liver function should be monitored, and the patients should be educated about the symptoms and signs of the hepatotoxicity. Isoniazid, pyrazinamide, and ethambutol are potentially hepatotoxic. They are all metabolized in the liver and might interact with each other as well as other drugs, leading to a higher risk of hepatotoxicity. In our case, hepatotoxicity was resolved after discontinuation of all the anti-TB medications. Pyrazinamide seems to be the most hepatotoxic agent in the quadruple regimen we chose, while the risk of isoniazid-induced hepatitis seems to be lower than previously thought.^14^ We restarted isoniazid/rifampin, but hepatitis recurred. After the resolution of hepatitis, we empirically started the combined isoniazid/ethambutol regimen. Hepatitis did not recur with this new regimen, and we continued it for 18 months. Rifampin or isoniazid/rifampin combination was suspected as the cause of hepatitis in our patient. Clinical response to the combined isoniazid/ethambutol regimen was favorable and his vision was considerably improved two months after initiating the treatment.

## Conclusion

Ocular TB is a great mimicker of various forms of intraocular inflammation. Our case showed that we should suspect TB in any case with intraocular inflammation, particularly in TB-endemic areas. Systemic signs and symptoms encourage us to search for a primary source of TB, whether pulmonary or extrapulmonary. A medical team consisting of an ophthalmologist and an infectious disease specialist might be needed for the diagnosis and management of ocular TB.
